# Supplementary material for: Association between Heated Tobacco Product Use during Pregnancy and Fetal Growth in Japan: A Nationwide Web-Based Survey
Source: Int J Environ Res Public Health. 2022 Sep 19;19(18):11826. doi: 10.3390/ijerph191811826 (PMC9517232; doi:10.3390/ijerph191811826)
Supplement: Supplementary file 1 [file ijerph-19-11826-s001.zip › ijerph-1895831-supplementary.pdf]

Association between heated tobacco product use during pregnancy and fetal growth in Japan: A nationwide web-based survey

Supplementary Material

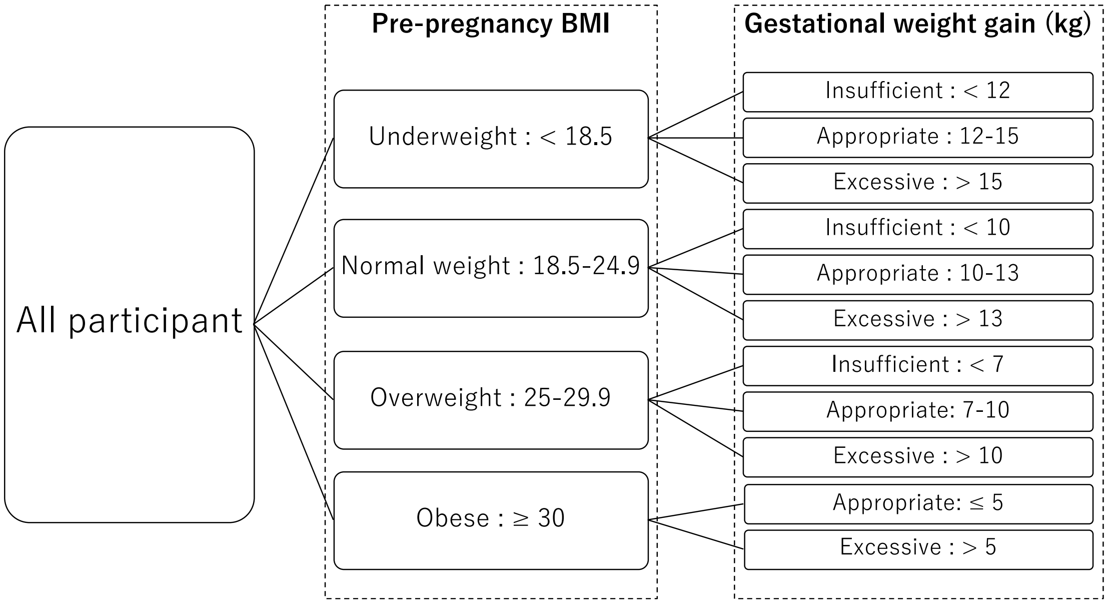

Figure S1. Categories of postdelivery women based on pre-pregnancy BMI and weight gain during pregnancy
The participants were classified into the following four groups according to their pre-pregnancy BMI: underweight, normal weight, overweight, and obese. Subsequently, the weight gain during pregnancy was classified into the following groups based on the appropriate weight gain during pregnancy according to the pre-pregnancy BMI: insufficient, appropriate, and excessive.
BMI, body mass index

Table S1. Prefecture distribution of participants, all postpartum women in 2020, and Japanese women between the ages of 20 and 50 years

	**Study participants n = 5,682**		**Japanese postpartum women in 2020[a] n = 840,804**		**Japanese women between the ages of 20 and 50 years[b] n = 21,385,480**	
	**N**	**%**	**N**	**%**	**N**	**%**
Hokkaido	215	3.8	29,523	3.5	852,270	4.0
Aomori	43	0.8	6,837	0.8	186,850	0.9
Iwate	22	0.4	6,718	0.8	180,134	0.8
Miyagi	113	2.0	14,480	1.7	395,843	1.9
Akita	28	0.5	4,499	0.5	133,611	0.6
Yamagata	36	0.6	6,217	0.7	157,451	0.7
Fukushima	59	1.0	11,215	1.3	283,832	1.3
Ibaraki	88	1.5	17,389	2.1	457,670	2.1
Tochigi	59	1.0	11,807	1.4	311,780	1.5
Gunma	60	1.1	11,660	1.4	303,765	1.4
Saitama	330	5.8	47,327	5.6	1,278,916	6.0
Chiba	242	4.3	40,168	4.8	1,079,368	5.0
Tokyo	675	11.9	99,661	11.9	2,732,942	12.8
Kanagawa	400	7.0	60,865	7.2	1,637,391	7.7
Niigata	87	1.5	12,980	1.5	339,664	1.6
Toyama	55	1.0	6,256	0.7	160,076	0.7
Ishikawa	49	0.9	7,712	0.9	185,127	0.9
Fukui	29	0.5	5,313	0.6	119,996	0.6
Yamanashi	31	0.5	5,184	0.6	124,586	0.6
Nagano	81	1.4	12,864	1.5	314,660	1.5
Gifu	111	2.0	12,092	1.4	311,264	1.5
Shizuoka	157	2.8	22,497	2.7	571,764	2.7
Aichi	470	8.3	55,613	6.6	1,296,231	6.1
Mie	66	1.2	11,141	1.3	280,724	1.3
Shiga	68	1.2	10,437	1.2	241,726	1.1
Kyoto	129	2.3	16,440	2.0	433,516	2.0
Osaka	521	9.2	61,878	7.4	1,592,330	7.4
Hyogo	295	5.2	36,952	4.4	940,222	4.4

Nara	61	1.1	7,831	0.9	220,184	1.0
Wakayama	40	0.7	5,732	0.7	145,974	0.7
Tottori	30	0.5	3,783	0.4	85,885	0.4
Shimane	30	0.5	4,473	0.5	97,532	0.5
Okayama	90	1.6	13,521	1.6	311,301	1.5
Hiroshima	168	3.0	19,606	2.3	462,122	2.2
Yamaguchi	60	1.1	8,203	1.0	201,415	0.9
Tokushima	24	0.4	4,521	0.5	112,767	0.5
Kagawa	36	0.6	6,179	0.7	155,547	0.7
Hiroshima	62	1.1	8,102	1.0	210,105	1.0
Kochi	19	0.3	4,082	0.5	104,624	0.5
Fukuoka	248	4.4	38,967	4.6	911,395	4.3
Saga	32	0.6	6,004	0.7	130,220	0.6
Nagasaki	40	0.7	9,182	1.1	201,256	0.9
Kumamoto	55	1.0	13,011	1.5	275,514	1.3
Oita	45	0.8	7,582	0.9	174,646	0.8
Miyazaki	34	0.6	7,719	0.9	166,417	0.8
Kagoshima	58	1.0	11,638	1.4	248,916	1.2
Okinawa	31	0.5	14,943	1.8	265,951	1.2

[a] Data on postpartum women in 2020 stratified by prefecture were extracted from government data.
[b] Data for women aged between 20 and 50 years stratified by prefecture were extracted from government data.

Table S2. Maternal characteristics of participants in this analysis

	N (%)			
	**Lifetime never smokers (N = 4,144)**	**Former smokers (N = 1,274)**	**Sole HTP smokers (N = 102)**	**Sole combustion smokers (N = 127)**
Delivery date				
July 2019–February 2020	553 (13.3)	156 (12.2)	14 (13.7)	25 (19.7)
March 2020–August 2020	1,020 (24.6)	335 (26.3)	29 (28.4)	29 (22.8)
September 2020–February 2021	1,507 (36.4)	475 (37.3)	31 (30.4)	48 (37.8)
March 2021–August 2021	1,064 (25.7)	308 (24.2)	28 (27.5)	25 (19.7)
Maternal age at survey				
< 25 years	106 (2.6)	48 (3.8)	9 (8.8)	10 (7.9)
25–29 years	1,122 (27.1)	281 (22.1)	37 (36.3)	31 (24.4)
30–34 years	1,753 (42.3)	476 (37.4)	35 (34.3)	33 (26.0)
35–39 years	985 (23.8)	390 (30.6)	18 (17.6)	44 (34.6)
≥ 40 years	178 (4.3)	79 (6.2)	3 (2.9)	9 (7.1)
Nulliparity	2,282 (55.1)	635 (49.8)	56 (54.9)	64 (50.4)
Pre-pregnancy BMI (kg/m²)				
< 18.5	796 (19.2)	212 (16.6)	27 (26.5)	19 (15.0)
18.5–24.9	2,952 (71.2)	895 (70.3)	63 (61.8)	87 (68.5)
25–29.9	235 (5.7)	107 (8.4)	7 (6.9)	16 (12.6)
≥ 30	161 (3.9)	60 (4.7)	5 (4.9)	5 (3.9)
Gestational weight gain (GWG)				
Pre-pregnancy BMI: underweight				
Insufficient: <,12 kg	628 (15.2)	143 (11.2)	13 (12.7)	13 (10.2)
Appropriate: 15 kg ≥ GWG ≥ 12 kg	128 (3.1)	51 (4.0)	9 (8.8)	4 (3.1)
Excessive: > 15 kg	40 (1.0)	18 (1.4)	5 (4.9)	2 (1.6)
Pre-pregnancy BMI: normal weight				
Insufficient: < 10 kg	1,589 (38.3)	401 (31.5)	24 (23.5)	31 (24.4)
Appropriate: 13 kg ≥ GWG ≥ 10 kg	1,036 (25.0)	333 (26.1)	19 (18.6)	28 (22.0)
Excessive: > 13 kg	327 (7.9)	161 (12.6)	20 (19.6)	28 (22.0)

Pre-pregnancy BMI: overweight				
Insufficient: < 7 kg	114 (2.8)	51 (4.0)	2 (2.0)	8 (6.3)
Appropriate: 10 kg ≥ GWG ≥ 7 kg	72 (1.7)	36 (2.8)	1 (1.0)	6 (4.7)
Excessive: > 10 kg	49 (1.2)	20 (1.6)	4 (3.9)	2 (1.6)
Pre-pregnancy BMI: obese				
Appropriate: ≤ 5 kg	54 (1.3)	30 (2.4)	3 (2.9)	1 (0.8)
Excessive: > 5 kg	107 (2.6)	30 (2.4)	2 (2.0)	4 (3.1)
In vitro fertilization	273 (6.6)	100 (7.8)	1 (1.0)	6 (4.7)
Smoking during the first trimester	0 (0.0)	0 (0.0)	95 (93.1)	121 (95.3)
Smoking during the second and third trimesters	0 (0.0)	0 (0.0)	44 (43.1)	49 (38.6)
Passive smoking everyday	299 (7.2)	213 (16.7)	64 (62.7)	59 (46.5)
Educational attainment ≥ 13 years	3,636 (87.7)	970 (76.1)	60 (58.8)	75 (59.1)
Married	4,099 (98.9)	1,254 (98.4)	98 (96.1)	117 (92.1)
Maternal complication				
Chronic hypertension	208 (5.0)	84 (6.6)	5 (4.9)	11 (8.7)
Diabetes	114 (2.8)	46 (3.6)	6 (5.9)	7 (5.5)
Chronic kidney disease	15 (0.4)	3 (0.2)	3 (2.9)	1 (0.8)
Autoimmune disease	39 (0.9)	13 (1.0)	3 (2.9)	2 (1.6)
Household income per year				
< 5 million JPY	907 (21.9)	356 (27.9)	29 (28.4)	46 (36.2)
5 to < 8 million JPY	1,342 (32.4)	428 (33.6)	28 (27.5)	34 (26.8)
≥ 8 million JPY	1,618 (39.0)	434 (34.1)	36 (35.3)	39 (30.7)
Declined to answer or do not know	277 (6.7)	56 (4.4)	9 (8.8)	8 (6.3)
Manager	363 (8.8)	84 (6.6)	8 (7.8)	13 (10.2)
Living in a prefecture with > 5 million population	2,486 (60.0)	741 (58.2)	77 (75.5)	76 (59.8)

BMI, body mass index; HTPs, heated tobacco products; JPY, Japanese yen

Table S3. Odds ratios for small for gestational age among former smokers, sole HTP smokers, and sole combustion smokers estimated using unconditional logistic regression

	Odds ratio (95% confidence interval)		
	**Model 1[a]**	**Model 2[b]**	**Model 3[c]**
Smoking status during pregnancy			
Lifetime never smokers	Reference	Reference	Reference
Former smokers	1.29 (0.89–1.87)	1.24 (0.85–1.87)	1.23 (0.84–1.79)
Sole HTP smokers	2.68 (1.13–6.36)	2.49 (1.04–5.97)	2.50 (1.03–6.05)
Sole combustion smokers	2.13 (0.90–5.03)	1.96 (0.82–4.69)	1.95 (0.81–4.67)
Maternal age at survey			
< 25 years	1.62 (0.66–3.99)	1.46 (0.59–3.63)	1.44 (0.58–3.59)
25–29 years	Reference	Reference	Reference
30–34 years	1.54 (1.00–2.36)	1.56 (1.02–2.41)	1.60 (1.03–2.46)
35–39 years	1.42 (0.88–2.28)	1.45 (0.90–2.34)	1.43 (0.89–2.32)
≥ 40 years	2.17 (1.09–4.31)	2.19 (1.09–4.37)	2.05 (1.02–4.11)
Pre-pregnancy BMI, Gestational weight gain			
PreBMI: underweight, GWG: insufficient	3.98 (2.37–6.67)	3.98 (2.37–6.68)	4.15 (2.46–6.98)
PreBMI: underweight, GWG: appropriate	1.32 (0.45–3.89)	1.31 (0.44–3.85)	1.35 (0.46–4.00)
PreBMI: underweight, GWG: excessive	0.97 (0.13–7.32)	0.93 (0.12–7.06)	0.99 (0.13–7.51)
PreBMI: normal weight, GWG: insufficient	2.28 (1.41–3.71)	2.26 (1.39–3.67)	2.31 (1.42–3.75)
PreBMI: normal weight, GWG: appropriate	Reference	Reference	Reference
PreBMI: normal weight, GWG: excessive	1.00 (0.45–2.19)	0.98 (0.44–2.15)	0.95 (0.43–2.10)
PreBMI: overweight, GWG: insufficient	1.03 (0.30–3.47)	0.99 (0.29–3.35)	0.90 (0.27–3.07)
PreBMI: overweight, GWG: appropriate	0.50 (0.07–3.75)	0.48 (0.06–3.63)	0.42 (0.06–3.20)
PreBMI: overweight, GWG: excessive	1.60 (0.37–6.97)	1.48 (0.34–6.49)	1.22 (0.28–5.41)
PreBMI: obese, GWG: appropriate	0.68 (0.09–5.14)	0.68 (0.09–5.10)	0.64 (0.09–4.84)
PreBMI: obese, GWG: excessive	1.33 (0.39–4.49)	1.30 (0.38–4.42)	1.27 (0.37–4.33)
Household income per year			
< 5 million JPY		1.21 (0.79–1.85)	1.20 (0.79–1.84)
5 to < 8 million JPY		Reference	Reference
≥ 8 million JPY		1.14 (0.77–1.69)	1.16 (0.78–1.72)
Declined to answer or do not know		1.45 (0.77–2.74)	1.46 (0.77–2.76)
Manager			

Yes	0.81 (0.43–1.51)	0.80 (0.42–1.49)
No	Reference	Reference
Education attainment		
≥ 13 years	Reference	Reference
< 13 years	1.32 (0.87–2.00)	1.31 (0.86–1.99)
Live in prefecture with > 5 million population		
Yes	Reference	Reference
No	1.17 (0.85–1.60)	1.17 (0.85–1.62)
Date of delivery		
July 2019–February 2020	Reference	Reference
March 2020–August 2020	1.18 (0.70–2.01)	1.15 (0.68–1.96)
September 2020–February 2021	1.14 (0.68–1.89)	1.10 (0.66–1.84)
March 2021–August 2021	1.01 (0.58–1.76)	0.99 (0.57–1.72)
Hypertensive disorders of pregnancy		
Yes		2.32 (1.44–3.74)
No		Reference
Chronic kidney disease		
Yes		1.62 (0.19–13.96)
No		Reference
Autoimmune diseases		
Yes		0.44 (0.05–3.67)
No		Reference

BMI, body mass index; GWG, gestational weight gain; HTPs, heated tobacco products; JPY, Japanese yen; PreBMI, pre-pregnancy body mass index; SGA, small for gestational age

[a] Multiple logistic regression adjusted for maternal age, pre-pregnancy body mass index, and gestational weight gain.

[b] Additional adjustment for household income, occupation, education attainment, living in a prefecture with >5 million population, and date of delivery.

[c] Additional adjustment for hypertensive disorders of pregnancy, chronic kidney disease, and autoimmune diseases.

Table S4. Odds ratios for small for gestational age among lifetime never smokers, sole HTP smokers (< 10 per day), and sole HTP smokers (≥ 10 per day) estimated using unconditional logistic regression

	Odds ratio (95% confidence interval)		
	Model 1[a]	Model 2[b]	Model 3[c]
Smoking status during pregnancy			
Lifetime never smokers	Reference	Reference	Reference
sole HTP smokers < 10 per day	1.77 (0.41–7.64)	1.58 (0.36–6.94)	1.45 (0.32–6.67)
sole HTP smokers ≥ 10 per day	3.48 (1.20–10.09)	3.14 (1.06–9.30)	3.14 (1.06–9.30)
Maternal age at survey			
< 25 years	1.48 (0.50–4.34)	1.35 (0.45–4.02)	1.44 (0.48–4.28)
25–29 years	Reference	Reference	Reference
30–34 years	1.34 (0.84–2.15)	1.37 (0.85–2.21)	1.41 (0.87–2.26)
35–39 years	0.93 (0.52–1.65)	0.96 (0.54–1.71)	0.95 (0.53–1.70)
≥ 40 years	2.29 (1.05–5.01)	2.31 (1.05–5.08)	2.08 (0.94–4.63)
Pre-pregnancy BMI, Gestational weight gain			
PreBMI: underweight, GWG: insufficient	5.72 (2.89–11.33)	5.74 (2.90–11.39)	6.04 (3.04–12.01)
PreBMI: underweight, GWG: appropriate	1.30 (0.28–5.96)	1.28 (0.28–5.86)	1.38 (0.30–6.36)
PreBMI: underweight, GWG: excessive	1.98 (0.25–15.92)	1.86 (0.23–15.13)	1.96 (0.24–15.94)
PreBMI: normal weight, GWG: insufficient	3.26 (1.70–6.27)	3.20 (1.66–6.15)	3.33 (1.73–6.42)
PreBMI: normal weight, GWG: appropriate	Reference	Reference	Reference
PreBMI: normal weight, GWG: excessive	1.28 (0.44–3.72)	1.25 (0.43–3.64)	1.23 (0.42–3.63)
PreBMI: overweight, GWG: insufficient	2.49 (0.68–9.09)	2.41 (0.66–8.84)	2.24 (0.61–8.27)
PreBMI: overweight, GWG: appropriate	1.26 (0.16–9.96)	1.22 (0.16–9.67)	1.15 (0.14–9.15)
PreBMI: overweight, GWG: excessive	1.60 (0.20–12.91)	1.52 (0.19–12.36)	1.34 (0.17–10.89)
PreBMI: obese, GWG: appropriate	1.53 (0.19–12.13)	1.56 (0.20–12.39)	1.40 (0.17–11.20)
PreBMI: obese, GWG: excessive	1.69 (0.37–7.73)	1.65 (0.36–7.63)	1.64 (0.36–7.57)
Household income per year			
< 5 million JPY		1.23 (0.73–2.07)	1.23 (0.73–2.07)
5 to < 8 million JPY		Reference	Reference
≥ 8 million JPY		1.21 (0.76–1.93)	1.25 (0.78–1.99)
Declined to answer or do not know		1.43 (0.69–2.98)	1.46 (0.70–3.04)
Manager			
Yes		0.66 (0.30–1.44)	0.66 (0.30–1.45)

No	Reference	Reference
Education attainment		
≥ 13 years	Reference	Reference
< 13 years	1.43 (085–2.41)	1.45 (0.86–2.44)
Live in prefecture with > 5 million population		
Yes	Reference	Reference
No	1.00 (0.68–1.46)	1.01 (0.69–1.48)
Date of delivery		
July 2019–February 2020	Reference	Reference
March 2020–August 2020	1.40 (0.71–2.77)	1.35 (0.68–2.67)
September 2020–February 2021	1.52 (0.80–2.92)	1.46 (0.76–2.80)
March 2021–August 2021	1.22 (0.61–2.45)	1.18 (0.59–2.37)
Hypertensive disorders of pregnancy		
Yes		2.44 (1.36–4.35)
No		Reference
Chronic kidney disease		
Yes		3.51 (0.43–28.65)
No		Reference

BMI, body mass index; GWG, gestational weight gain; HTPs, heated tobacco products; JPY, Japanese yen; PreBMI, pre-pregnancy body mass index;

[a] Multiple logistic regression adjusted for maternal age, pre-pregnancy body mass index, and gestational weight gain.

[b] Additional adjustment for household income, occupation, education attainment, living in a prefecture with over 5 million population, and date of delivery.

[c] Additional adjustment for hypertensive disorders of pregnancy, and chronic kidney disease.
